# Real-time Brain Tumor imaging with endogenous fluorophores: a diagnosis proof-of-concept study on fresh human samples

**DOI:** 10.1038/s41598-018-33134-2

**Published:** 2018-10-05

**Authors:** Fanny Poulon, Johan Pallud, Pascale Varlet, Marc Zanello, Fabrice Chretien, Edouard Dezamis, Georges Abi-Lahoud, François Nataf, Baris Turak, Bertrand Devaux, Darine Abi Haidar

**Affiliations:** 1 IMNC Laboratory, UMR 8165-CNRS/IN2P3, Paris-Saclay university, 91405 Orsay, France; 2 Neurosurgery Department, Sainte-Anne Hospital, Paris, France; 3 Neuropathology Department, Sainte-Anne Hospital, Paris, France; 4 IMA BRAIN, INSERMU894, Centre de Psychiatrie et de Neurosciences, Paris, France; 5 Paris Descartes University, Paris, France; 6 Paris Diderot University, Sorbonne Paris Cité, F-75013 Paris, France

## Abstract

The primary line of therapy for high-grade brain tumor is surgical resection, however, identifying tumor margins *in vivo* remains a major challenge. Despite the progress in computer-assisted imaging techniques, biopsy analysis remains the standard diagnostic tool when it comes to delineating tumor margins. Our group aims to answer this challenge by exploiting optical imaging of endogenous fluorescence in order to provide a reliable and reproducible diagnosis close to neuropathology. In this study, we first establish the ability of two-photon microscopy (TPM) to discriminate normal brain tissue from glioblastomas and brain metastasis using the endogenous fluorescence response of fresh human brain sample. Two-photon fluorescence images were compared to gold standard neuropathology. “Blind” diagnosis realized by a neuropathologist on a group of TPM images show a good sensitivity, 100%, and specificity, 50% to discriminate non tumoral brain tissue versus glioblastoma or brain metastasis. Quantitative analysis on spectral and fluorescence lifetime measurements resulted in building a scoring system to discriminate brain tissue samples.

## Introduction

Surgical resection aims to maximize tumor removal while minimizing morbidity for both primary and metastatic brain tumors^1,2^. Such an approach requires the identification of the surgical margins that can be defined by the limits of normal tissue and/or the extent of tumor infiltration. For normal tissue, particularly eloquent areas, that represent the corticosubcortical functional pathways, need to be preserved. Even if eloquent cortico-subcortical pathways can be identified intraoperatively using brain mapping with direct electrical stimulations under awake conditions^3,4^, the identification of tumor boundaries remains challenging. Nowadays, tumor margins are identified based on the neurosurgeon’s experience and with the aid of operating microscopes, MRI-based and/or ultrasonography-based neuronavigation, and intraoperative MRI^5^. Unfortunately, none of these techniques have sufficient spatial resolution to identify tumor infiltration at the cellular level and to discriminate infiltrating tumor from surgically-induced brain tissue alterations (i.e. contusion, ischemia or edema)^6,7^. The assessment of tumor borders at the cellular scale can be performed using pathological intraoperative examination, however this technique is not adapted for use during surgery due to time and sampling technique constraints.

The inspection of endogenous brain fluorophores, such as reduced Nicotinamide Adenine Dinucleotide (NADH), Flavin Adenine Dinucleotide (FAD), lipopigments, and porphyrins I and II, all of which considered as biomarkers of cell energy metabolism^8,9^, is a promising key to perform optical imaging at the cellular scale. Interestingly, by examining tissue autofluorescence, one is avoiding prejudice that results from the use of external markers, such as 5-Aminolevulinic Acid (5-ALA) that enhances protoporphyrin IX (PpIX) fluorescence. In addition, although such markers induce fluorescence, they do so through molecular links or processes that are not natural and could therefore result in artifact in the fluorescence response. Evaluating intrinsic optical signals using two photon microscopy (TPM) gives access to two imaging contrasts; fluorescence and second harmonic generation, which both act as complementary modalities giving high resolved spatial information. This has motivated its use in real-time optical biopsy in different cancer types, such as breast tumor masses^10^, liver cancer^11^ or even pancreatic cancer^12^. Moreover, the ability to combine this technique to quantitative measurements such as spectroscopy and fluorescence lifetime, results in reliable and reproducible discrimination in clinical settings based on an endogenous contrast. Compared to other endomicroscopy techniques, such as confocal laser endoscope, TPM provides multiple advantages^13^ such as (1) intrinsic sectioning up to 1 mm; (2) no out-of-focus photobleaching and photodamage; (3) localized phototoxicity and photobleaching; (4) deeper penetration into biological tissue when set side by side with confocal microscopy (up to 1 mm)^14^, and (5) no spectral overlapping between excitation and emission signals. To top it up, four different optical contrast mechanisms which are: (1) spectral analysis; (2) two photon Fluorescence Lifetime Imaging Microscopy (FLIM); (3) Second Harmonic Generation (SHG) imaging^15^, and (4) Two Photon Excitation Fluorescence (TPEF), can be extracted with the use of TPM to provide complementary information for improved tissue characterization.

The aim of the present study was to evaluate the ability of TPM in differentiating tumorous brain tissue from normal tissue in order to support the development of an intraoperative two-photon endomicroscope, that will be able to give a real-time answer to the surgeon. We investigated TPM autofluorescence signal analysis from the visible to the infrared domains, exploring all known endogenous molecules coming from freshly extracted brain tissue, including normal (control) tissue, glioblastoma (GBM), and brain metastasis collected from adult patients. In this study, we evaluated: (1) the capacity of TPM in distinguishing between tumorous and normal tissue; (2) the correlation between the optical signatures extracted from TPM and the histopathological diagnosis derived from the gold standard, whereby we can evaluate the clinical relevance of TPM as a more robust intraoperative diagnosis modality, and (3) the predictive power of TPM imaging features in differentiating malignant gliomas from normal tissue as well as from brain metastasis.

## Results

The routines of the TPM procedure match the requirements for clinical use of freshly resected brain tissue. Specimens from twenty-five patients, comprising 10 metastasis, 8 GBM, and 7 epileptic patients with no history of brain tumors, were included in the cohort. Fresh samples were either sent to histopathology and/or to the multimodal imaging platform (PIMPA), providing similar imaging conditions for TPEF, SHG, FLIM and spectral imaging. In the histopathology circuit, the samples followed a first protocol of fixation, paraffin embodiment and hematoxylin and eosin (H&E) coloration that lasts several hours, then the stained samples were imaged and analyzed by neuropathologists. In the multimodal imaging platform circuit, the samples were directly placed (without any preparation or chemical modification) under the multiphoton microscope, from which a multimodal imaging protocol (TPEF/SHG, spectral, FLIM) was performed within several seconds, after which the results were analyzed.

Being a crucial point to take into account, the time required to obtain imaging data from brain samples was compared in each of the above circuits. The standard histological H&E staining method, that results in the most precise diagnostic and that yields the nature and the grading of the tumor, required 450 ± 30 min. Surgeons can also use a faster method during surgery, with the help of a neuropathologist, based on frozen sections that can be evaluated in 20 min in parallel to the operation and yield some information on the lesioned nature of tissue. However, both these techniques were more time consuming than the required time of 0.01 ± 0.005 min for the optical analysis method by TPM, which included data from TPEF, SHG, FLIM and spectral imaging (p < 0,001), with a real-time discriminating algorithm. Additionally, TPM has a major advantage over both histological techniques, the analysis could be in performed in the future on *in-vivo* and unlabeled tissue.

### TPM images match standard histopathology with H&E staining

The work then comprised a thorough comparison between the TPM based images and the H&E-stained images. The PIMPA platform provides simultaneously two different images obtained by the two external hybrid detectors: a TPEF image at the maximum fluorescence peak (filter 520 ± 20 nm) to capture tissue characteristics; and a SHG image (filter 448 ± 20 nm) to detect non centrosymmetric structures such as collagen fibers and vessels walls. The TPEF and SHG images were merged using ImageJ to obtain a superimposed image showing the complete morphological structures of the tissue.

**Figure 1:**
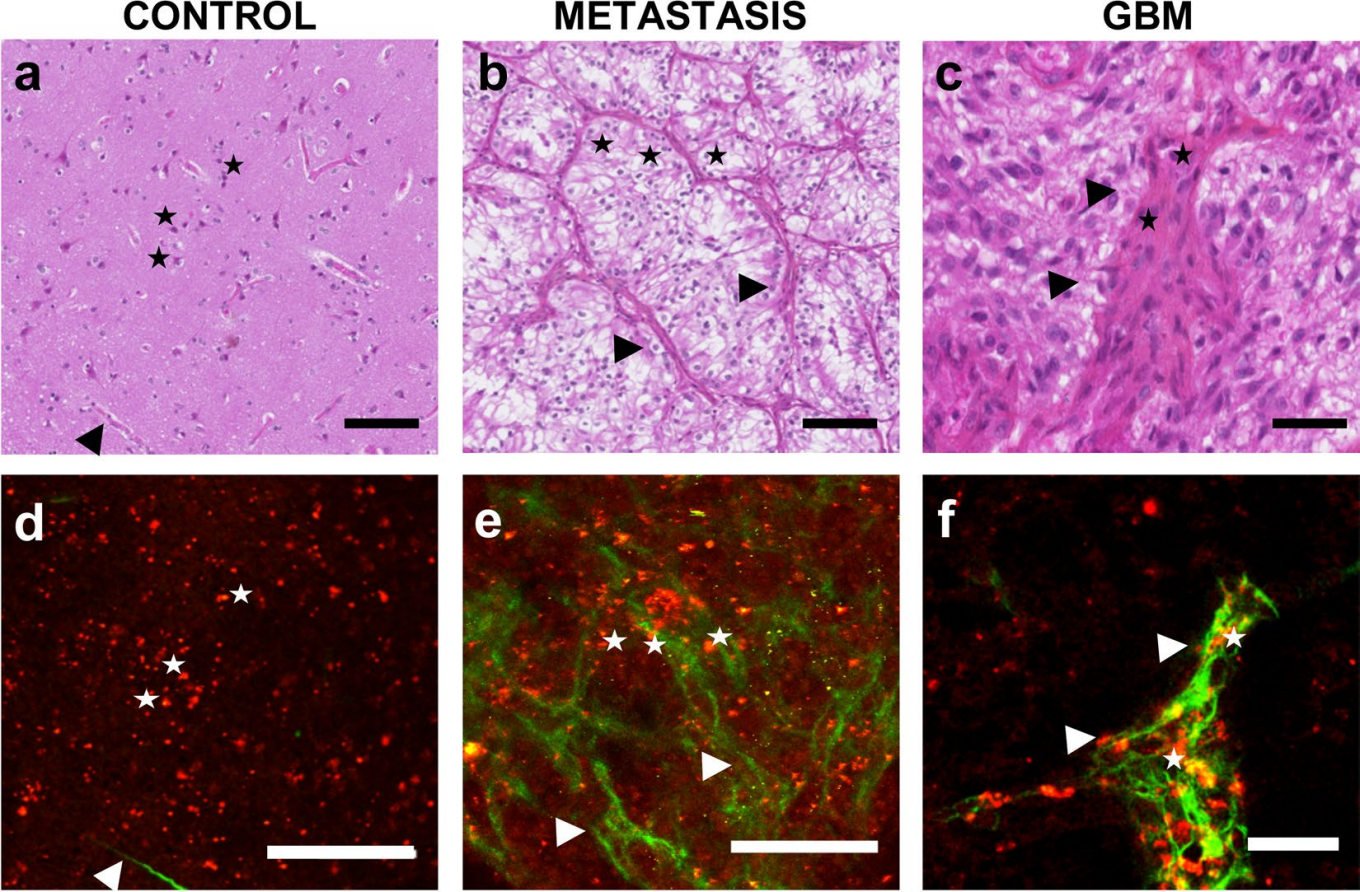
Comparative H&E (**a**–**c**) and TPM images (**d**–**f**). (**a,d**) Control sample, stars: neurons, arrow: brain vessels in the SHG, scale bar 100 microns. (**b,e**) Brain carcinoma metastasis: scale bar 100 microns, stars: tumor cells, arrow: dense vascularization forming a dense network around the tumor cells, (**c,f**) GBM sample scale bar 40 microns: stars: proliferative endothelial cells, arrow: zoom on a proliferating vessel.

For each sample, a senior neuropathologist was asked to identify normal or tumorous cellular components (neuron oligodendrocyte, astrocyte; endothelial cell, carcinoma cell) and/or a particular morphological aspect (necrosis, neo-angiogenesis and microvascular proliferation, calcification) on H&E-stained images that served as a reference, and was then asked to localize these patterns in the merged TPEF and SHG images. In all samples (n = 25), the senior neuropathologist was able to identify in TPEF and SHG images the features that were first identified on H&E-stained images. Examples are shown in Fig. 1.

In control brain samples, cellular nuclei appeared as hypointense structures in a homogeneous matrix-dominated background. Neuronal cells were easily discernable in both H&E stained images as well as merged TPEF-SHG images (black and white stars in Fig. 1a,b). In brain metastasis, typical cytoarchitecture hallmarks such as hypercellularity and disorganized stroma with numerous blood vessels generated a particular SHG signal (black and white arrows in Fig. 1c,d). In GBM samples, a highly cellular disorganized tumorous cell architecture was observed with microvascular proliferation. In general, the SHG signal highlights collagen structure with the metastasis samples having the densest collagen network. The control and GBM samples revealed sparse SHG signals representative of vessel walls.

### “Blind” analysis of TPM images

**Figure 2:**
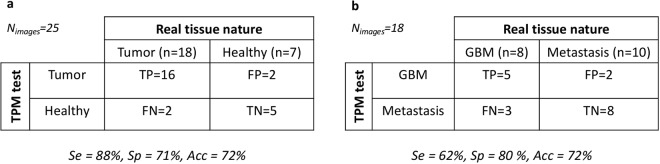
“Blind” histological analysis on TPM images. (**a**) Flowchart summarizing the results of the pathologists’ diagnosis of tumoral nature based on TPM images, sensitivity (Se), specificity (Sp) and accuracy (Acc) of this method were calculated. (**b**) Flowchart summarizing results of the pathologists’ discriminating GBM from brain metastasis. The sensitivity (Se), specificity (Sp) and accuracy (Acc) were also calculated.

We next assessed the clinical application of TPM imaging and its capacity to show specific patterns of the different brain tumors needed in clinical practice to discriminate them. Twenty-five TPM images (control, n = 7, GBM, n = 8, metastasis, n = 10) were given to neuropathologists blind to the nature of tissue. They were asked to classify the tissue in four categories: (1) GBM; (2) Brain metastasis; (3) Normal brain parenchyma or (4) impossible diagnosis. The neuropathologists were asked to use TPM derived markers, as determined in the previous section (cell, cell density, collagen fiber density and organization, angiogenesis, microvascular proliferation, and necrosis) to propose a diagnosis. The results are summarized in Fig. 2.

Discriminating tumorous tissue from control tissue using TPM resulted in a sensitivity of 88% and a specificity of 71%. The accuracy of this procedure, which means the probability to correctly classify tissues, was 72%. Interestingly, from the results of the neuropathologists, the ability to discriminate GBM tissues from metastasis tissues using TPM could also be evaluated. This resulted in a sensitivity of 62% and a specificity of 80%. The accuracy of this procedure, was found to be 72%.

### Quantitative analyses of TPM signals

**Figure 3:**
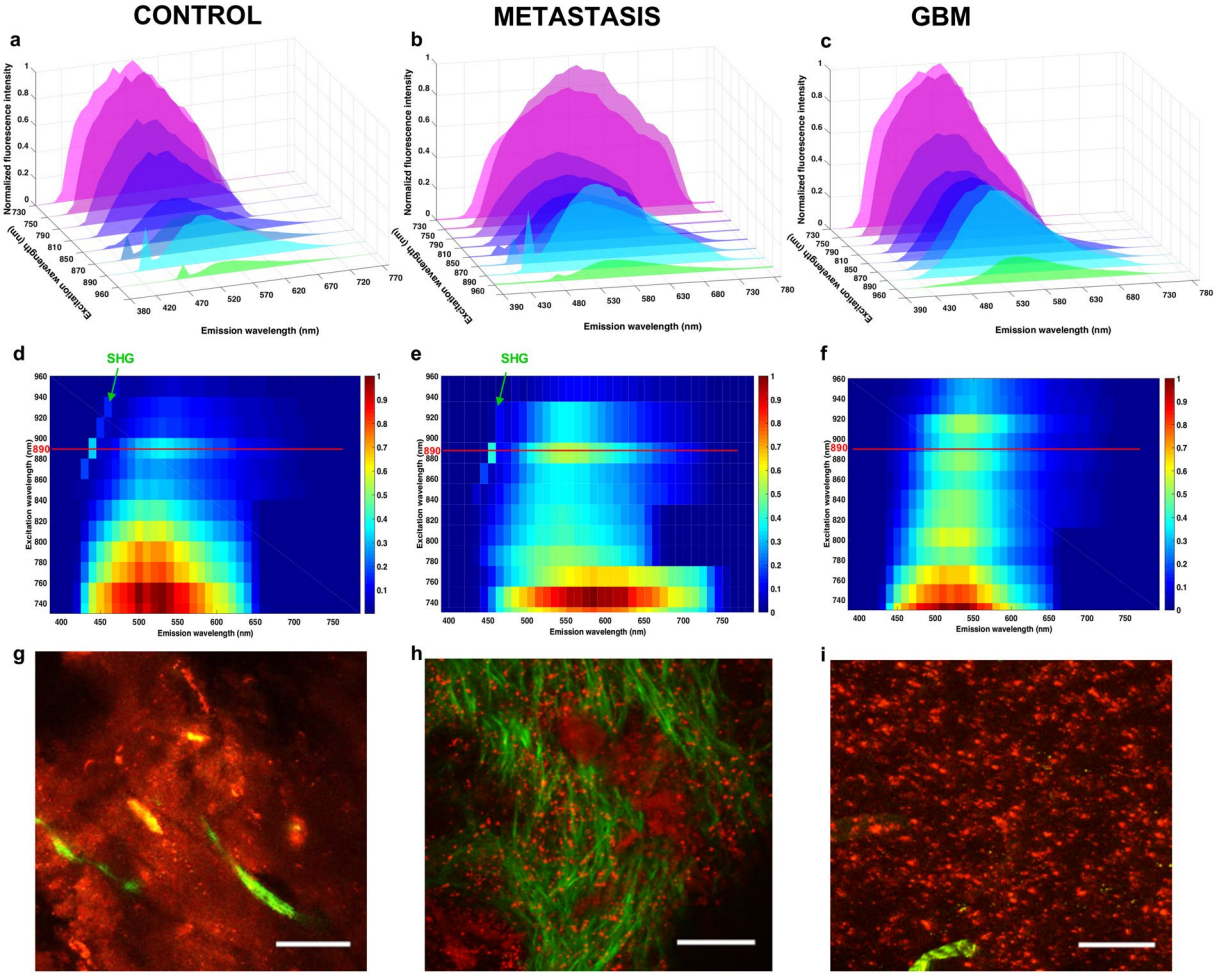
Analysis of emitted fluorescence for the different groups. (**a–c**) Topological representation of the emitted fluorescence spectra at different excitation wavelengths for the control (**a,d,g**), the metastasis (**b,e,h**) and the GBM (**c,f,i**) groups. (**d–f**) Represent colormaps of the Emission-Excitation matrix for each type of tissue. The ideal excitation wavelength is highlighted by a red line and the part of the map corresponding to the SHG signal is identified by a green arrow. TPEF images (**g–i**) of the selected region is also shown with a scale bar of 100 microns.

To determine reliable discriminating criteria, quantitative analyses of spectral and FLIM images were performed on each sample (n = 25; n = 10 metastasis, n = 8 GBM and n = 7 control). Different wavelengths (from 730 nm to 960 nm) were applied to the three subgroups to define the optimal excitation wavelength for collecting spectral and FLIM images as well as the SHG signals. The variation of endogenous fluorescence was measured in order to build an excitation-emission matrix for control, GBM, and metastasis samples. The results are presented in Fig. 3.

The fluorescence intensity decreased by 70% as the excitation wavelength changed from 730 to 900 nm. However, secondary maximal fluorescence intensity was detected at 890 nm (60% of the maximum intensity) in a region where SHG signal peaks. Control samples and GBM samples had a comparable decrease of 15% in the fluorescence intensity, when the excitation wavelength varied from 730 nm to 760 nm. Interestingly, metastasis samples had a faster rate of decrease (30%) in the fluorescence intensity. In all three subgroups, the overall emitted fluorescence intensities remained similar upon excitations ranging from 850 nm to 900 nm. The emission spectra then decreases when fluorophores are excited at longer wavelengths. This optimal 850–900 nm spectral range for excitation coincides with the emission spectral range optimized for SHG detection. Consequently, further TPM based imaging were conducted under an 890 nm excitation.

It is clear that the emission spectra are red-shifted when the longest excitation wavelengths are used. Additionally, fluorescence emitted around 470 nm, which is mainly attributed to NADH autofluorescence is greatly suppressed. This is explained by NADH’s suboptimal absorption cross section at wavelengths longer than 800 nm. FAD on the other hand showed a second maximal absorption cross section around 900 nm^16^.

**Figure 4:**
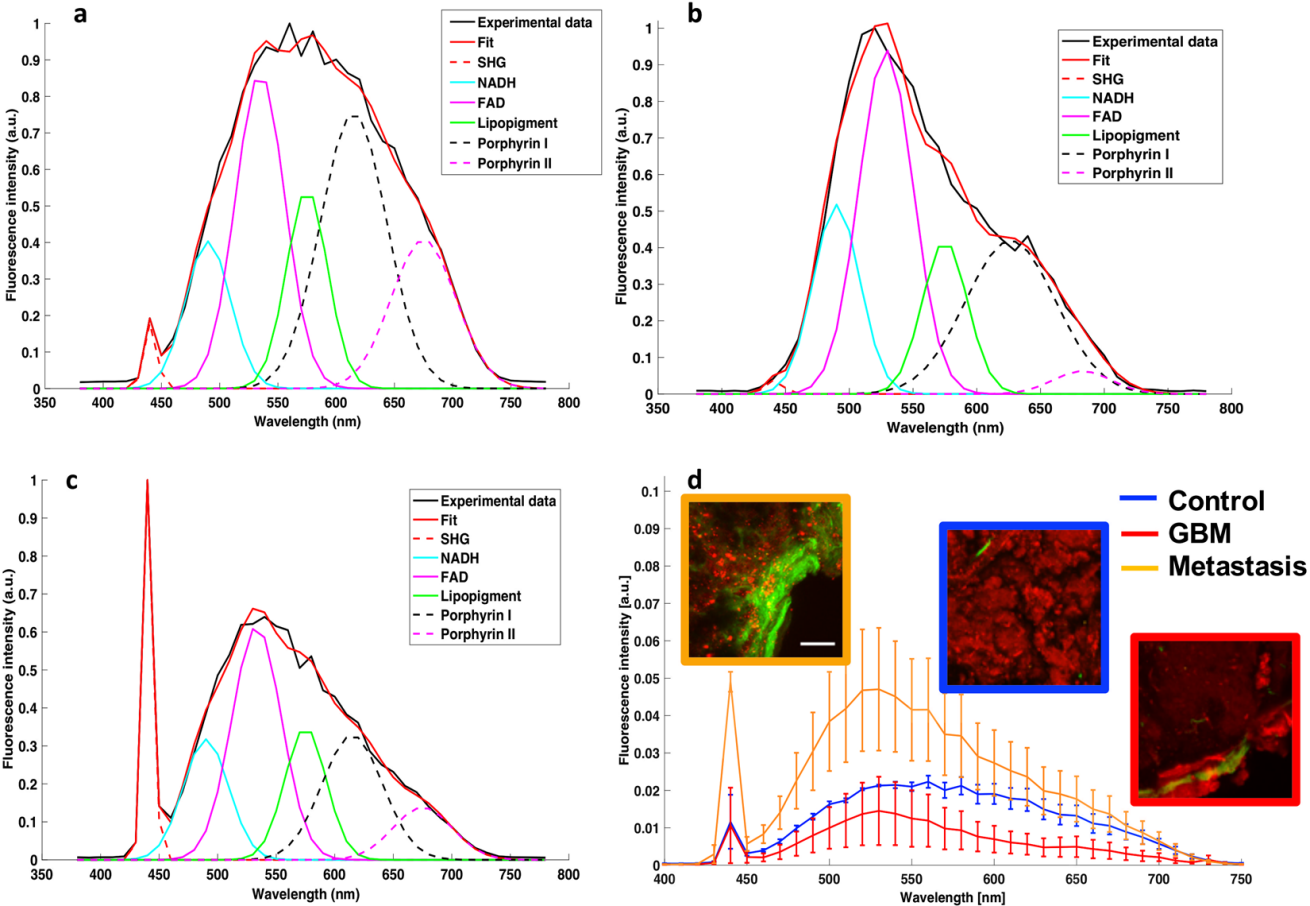
Spectral decomposition and comparison of emitted spectra at 890 nm for each group. (**a**–**c**) Example of fitted spectra for control (**a**), metastasis (**b**), and GBM (**c**), under 890 nm excitation. (**d**) Mean spectra and standard deviations determined from 25 fresh human samples (10 metastasis samples, 8 GBM samples and 7 control samples) along with representative images of TPEF (red) and SHG (green) corresponding to each tissue group.

In all cases, the spectral shape differed between subgroups indicating the differences in the relative amounts of individual fluorophores present in the three tissue groups. An example of the fitted spectra at 890 nm of each subgroup is shown in Fig. 4.

**Figure 5:**
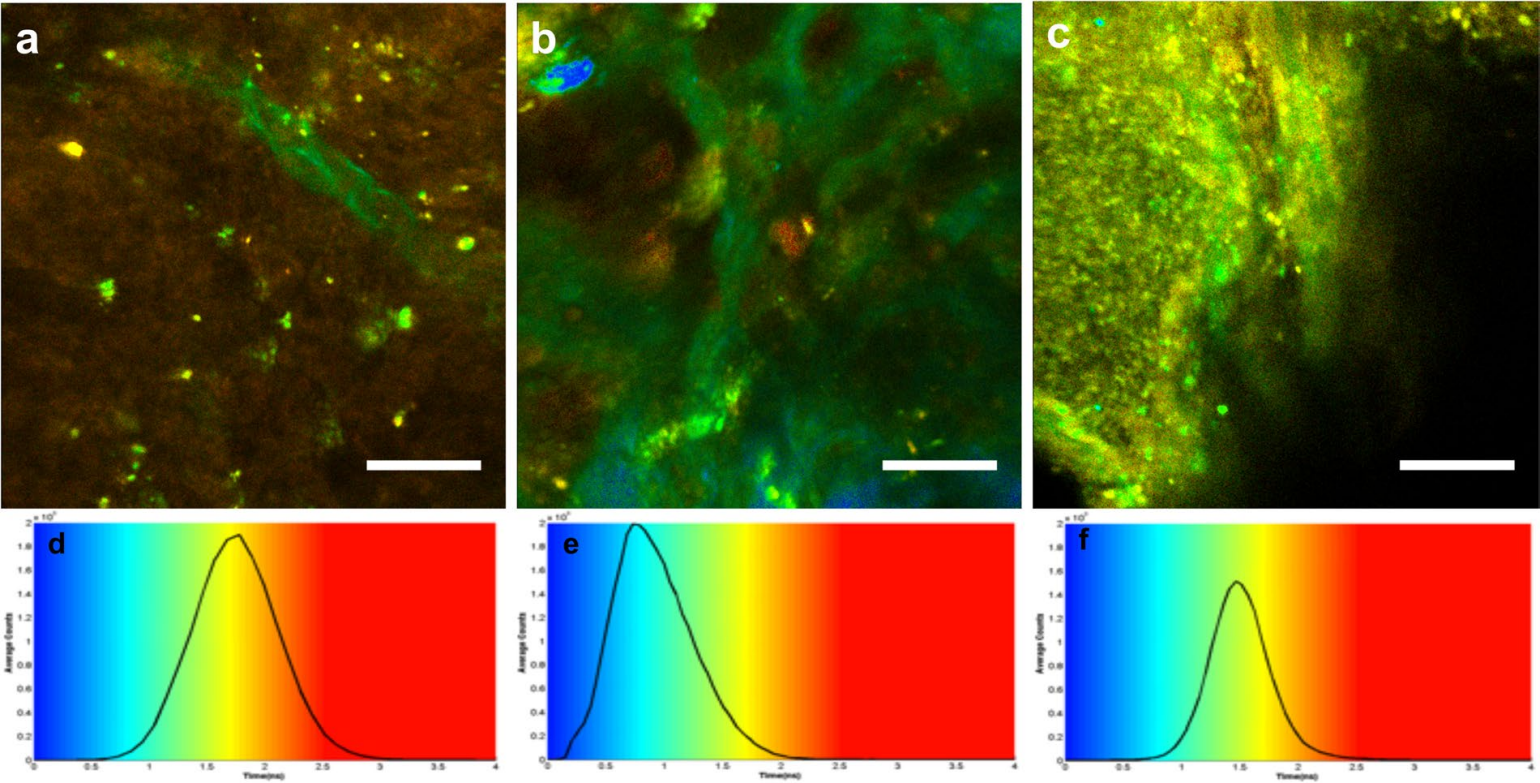
FLIM representation at 890 nm for each tissue type. (**a–c**) FLIM images of control sample (**a**), metastasis sample (**b**), and GBM (**c**). Scale bars of 100 μm, (**d**–**f**) give the color scale of FLIM imaging with the histogram of the average lifetime; shorter lifetimes (blue) are on the left side of the color scale, and longer lifetime (red) on the right side, two measurements were taken and the full width at half maximum of distribution and the average lifetime of the maximum. SHG is not a fluorescent but diffusing process, appearing as very short lifetime corresponding to the instrument response function (IRF), which is of the order of 0.06 ns and shown in blue in the FLIM images. The typical vascular structures of each tissue are consequently as recognizable as in the TPEF images.

Figure 4a–c show a spectrally decomposed fitted spectra (n = 1) for a sample from each tissue group. The fluorescence coming from five endogenous fluorophores was recovered, namely, NADH, FAD, lipopigments, porphyrins I and II. The SHG peaks were also extracted. The control samples (Fig. 5a) presented a broader fluorescence spectrum compared to both metastatic and GBM samples. Spectra from GBM and metastasis samples (Fig. 5b,c) were particularly dominated by FAD fluorescence followed by Porphyrin I.

The metastatic samples showed a uniquely high SHG peak corresponding to the presence of dense vessels network, as shown in the TPEF/SHG image, which was always significantly higher than that of healthy and GBM tissue (p < 0.001: SHG signal: M_metastasis_ = 0.057, M_GBM_ = 0.0069 and M_control_ = 0.0054). GBM and healthy tissues exhibited similar SHG peaks suggesting that both types have comparable microvascular density. GBM did show larger blood vessels, facilitating angiogenesis on the corresponding TPEF/SHG images. Moreover, the full width at half maximum, L, computed from the total fluorescence spectra of control samples were found to be significantly different from the ones extracted in Metastatic and GBM tissue (L_metastasis_ = 128 nm, L_GBM_ = 134 nm, L_control_ = 185 nm).

A summary of the spectral analysis at the reference excitation wavelength of 890 nm is presented in Fig. 4d. GBM samples exhibited an overall lower fluorescence intensity than control samples supposedly due to the necrotic areas in these samples. This was confirmed by the TPEF images showing a global darker background. In the emission range of 600 to 650 nm, the mean emitted fluorescence was significantly lower (p < 0.001) in the GBM samples as compared to control and metastasis samples (M_GBM_ = 0.0055, M_metastasis_ = 0.0221 and M_control_ = 0.0160). Metastasis samples exhibited an overall higher fluorescence intensity than control samples due to the higher tumor cell density as previously confirmed by the TPEF images showing a high density of bright red spot, identified as tumor cells. In the emission range of 500 to 550 nm, the mean emitted fluorescence was significantly higher (p < 0.001) in the metastasis samples as compared to control and GBM samples (M_metastasis_ = 0.0435, M_GBM_ = 0.0035 and M_control_ = 0.0045).

One limitation to spectral response studies prevails in identifying tissue samples, the intensity is dependent on the fluorophore concentration, which can be very low in peripheral tumor volumes and in tumor margins. To overcome such an obstacle, we studied fluorescence lifetime, which is a quantitative optical measurement that depends on environmental conditions such as pH, temperature, viscosity, and structural changes including molecular conformation and binding partners, but not on fluorophore concentration. We quantified the fluorescence lifetime on all samples (n = 25; n = 10 metastasis, n = 8 GBM and n = 7 Control) through the FLIM technique. Figure 5 shows representative results for each tissue group.

The control samples had the broadest FLIM distribution (FWHM_Control_ = 0.856 ns compared to FWHM_metastasis_ = 0.764 ns and FWHM_GBM_ = 0.557 ns) due to a heterogenous range of components with short and long lifetime values that were present in similar quantity. GBM samples had a narrow FLIM distribution; the blue-green structures represented neo-angiogenesis. The metastasis samples had the shortest average lifetime at maximum (τ_metastasis_ = 0,7344 ns compared to τ_GBM_ = 1,47 ns and τ_control_ = 1,78 ns) compared to the Control and GBM samples as a result of the presence of a dense vascular network. However, the tissue background color in between vessels corresponds to longer fluorescence lifetimes than that of GBM samples (FWHM_metastasis_ = 0,764 ns > FWHM_GBM_ = 0,557 ns).

### TPM signals scoring system

**Figure 6:**
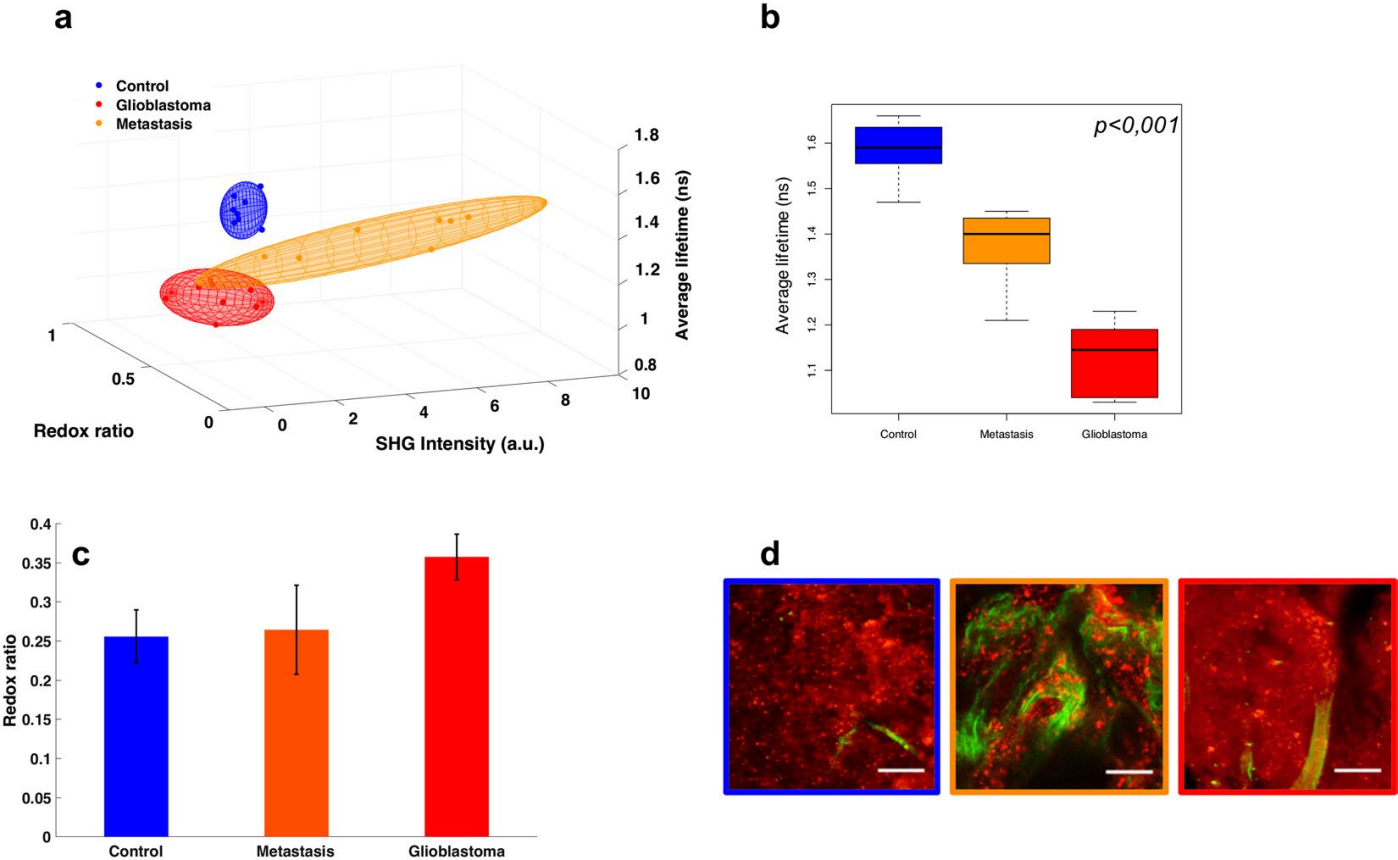
(**a**) 3D scatter plot of three quantitative tissue indicators: the redox ratio, fluorescence lifetime, and SHG signal averaged for each tissue subgroup with a Gaussian ellipsoid fit, (**b**) box plot of the average lifetimes, (**c**) bar graph of the redox ratios for each tissue type with the errors corresponding to the standard deviations across all measurements, and (**d**) overlaid TPEF and SHG intensity images.

To translate TPM imaging into the operating room, we aimed to develop quantitative parameters derived from the spectral analysis and the fluorescence lifetime imaging to construct a scoring system combining three quantitative tissue dependent variables: the redox ratio, the average lifetime and the SHG intensity. The redox ratio and the SHG intensity were calculated from the spectral fitting results whereas the average lifetime was calculated from the exponential-decay fit of the lifetime measurements. The three derived values were used as coordinates to plot each sample in a 3D space. Figure 6 summarizes these results.

Taken separately, each of the quantitative indicators were not always statistically significant while discriminating between the different tissue types. The average lifetime of both GBM samples and metastasis samples were notably shorter than those of Control samples (τ_GBM_ = 1.13 ns − τ_control_ = 1.59 ns, p < 0.001; τ_metastasis_ = 1.37 ns − τ_control_ = 1.59 ns, p < 0.001). In addition, the average lifetime of GBM samples was remarkably shorter than those of metastasis samples (τ_GBM_ = 1.13 ns − τ_metastasis_ = 1.37 ns, p < 0.001). On average, cancerous tissues had higher redox ratios (ROX_control_ = 0.256 ± 0.030, ROX_metastasis_ = 0.264 ± 0.030 and ROX_GBM_ = 0.357 ± 0.030), compared to normal tissues. Although it did not reach statistical significance, the larger trend was observed between GBM and control cases (ROX_control_ = 0.256, ROX_GBM_ = 0.357, p = 0.08). On the other hand, the SHG intensities were particularly helpful in discriminating metastatic samples from the remaining two types (SHG_metastasis_ = 3.8, τ_control_ = 0.35, p < 0.001 and SHG_metastasis_ = 3.8 − SHG_GBM_ = 0.46).

Consequently, in order to have a more vigorous mean of discrimination, these separate indicators were combined in a unique scoring system, Fig. 6a. The performances of the multimodal algorithm were generated using a Gaussian ellipsoid fit, where the control tissue never overlapped with any tumorous type in 95% confidence, and GBM-metastasis only had 16,6% of overlap.

## Discussion

In this prospective study, we compared TPM derived optical contrasts measured from different fresh human brain tumoral and non tumoral samples to gold standard neuropathology. We have demonstrated that: (1) TPM can readily be integrated into the operating room as the acquisition times are short; (2) The merged TPEF and SHG images showed some similar features as those observed by standard neuropathology particularly the vascular and stromal network (3) TPM imaging was capable of discriminating tumorous from normal tissue at a sensitivity and specificity of 88% and of 71% respectively. Interestingly, TPM also allowed the discrimination between GBM and metastasis tissues with a sensitivity of 62% and a specificity of 80%; (4) Quantitative TPM signals also categorized tissues according to their groups (control, GBM, metastasis); and lastly, (5) the combined scoring system, i.e. combining redox ratios, average lifetimes, and SHG intensities, allowed tissue samples to be discriminated, with no overlap between control and both GBM and metastasis response.

An important point to firstly stress on is the clinical benefit in acquiring TPEF in real-time compared to standard histological examination (t_TPM_ = 0.01 min < t_H&E_ = 450 min). A classical H&E image is time and labor consuming given the multiple steps and resting time that are required in order to obtain a stained slice. On the contrary, optical images are recorded in a few seconds without any tissue manipulation of the sample, samples being imaged freshly resected directly under the microscope. This point demonstrates the power of optical imaging in giving quick and highly resolved images as well as its ability to be used intraoperatively.

Second, having established the technical advantages of TPM, it was crucial to evaluate its capacity in discriminating tissues. Therefore, a typical comparison between TPEF-SHG images and histology gold standard H&E staining was performed, and a strong correlation was established between the two imaging modalities for each tissue type. The cortex could be identified by the presence of numerous neurons associated with thin branched vessels highlighted by SHG signal. Glioblastoma are characterized by a high cellularity and large glomerular vessels. Brain metastasis by contrast show the dense collagen vascular stroma, resulting in a very strong SHG signal.

The tissue specific signatures found in this comparison were used by a neuropathologist to perform a blind analysis of the TPM images. The statistical results of this diagnostic procedure gave promising results, with high sensitivity (between 75 and 100%) to discriminate tumor from control tissue, opening the doors to real-time optical biopsy. The low specificity (50%) of the technique can be improved by training the neuropathologist with these images as the standard practice for H&E.

A major common limitation between image comparison and H&E staining is the subjective diagnosis. For this reason, a real advantage of TPM is the combination of images with quantitative measurements to build a computed diagnosis that could be used directly *in vivo* by the surgeon. In this study, we explored two quantitative measurements, the spectral emission and the fluorescence lifetime to build a discriminative algorithm. The spectral measurement is sensitive to the nature and the concentration of fluorophores measured, thus giving access to information on the metabolic evolution of tissues, such as indicators of redox status by following NADH and FAD^8,9,17^. On the other hand the FLIM measurement is sensitive to binding states, molecular interferences, and other aspects of the molecular environment such as temperature, molecular liaisons, viscosity of the medium, and pH^18–22^. Therefore, TPM-FLIM data can provide important complementary information about the local biochemical medium that may aid in distinguishing healthy from tumorous tissues. Time-resolved fluorescence of endogenous response is a useful complementing tool that separates pathological tissues based on their metabolism^23,24^. The extra quantitative dimensions of information provided by spectral and lifetime imaging could facilitate diagnostic judgments. Consequently, three of the most significant quantitative indicators found in the exploratory analysis were combined to build a scoring system of brain tissue nature. These indicators were the redox ratio to monitor the metabolic state^17,23,25,26^, the SHG intensity to evaluate the density of collagen structures^21,27^, and the average fluorescence lifetime to track environmental and conformational changes^18–22^. Taken separately these numerical indicators gave us mixed results when discriminating tissues. For example, the Redox ratio failed to be robust, although it did discriminate GBM from control most accurately, with the tumors having higher ratios. This is in accordance with literature^25^ and is explained by the increase in tumorous metabolic needs (Warburg effect) resulting in changes in the NADH/FAD ratios^28^. The SHG intensity on the other hand gave us a statistical difference between control and metastasis, corresponding to the strong net of vessels and collagen matrix that form around the tumors cells as also referred to in the literature^29^. The average lifetime was the most robust classifier, where the values for tumor and control tissues were significantly different with a shorter lifetime found in tumor tissue. Nevertheless, when accounting for all three quantitative markers, as displayed in the 3D scatter plot of Fig. 6a, one could easily classify brain tissues. These results underline the necessity of developing an endomicroscope with multimodality capabilities for robust *in vivo* tissue interrogation.

All in all, these results based on tissue autofluorescence signals coming from brain tumors are taking part in the construction of an optical database that will be implemented in a two-photon multimodal endomicroscope. A first prototype of this intraoperative surgical tool is under development in our laboratory and has shown great performances when it comes to collecting signals^30,31^. Compared to the different diagnostic techniques such as frozen sections or formaldehyde fixed H&E stains performed by neuropathologists today, this method does not require any tissue resection. On the contrary, the probe can be directly put in contact with the human brain while performing a measurement. Another advantage is the turnaround time required to diagnose a tissue, which is estimated to be less than a minute from the moment the surgeon holds the probe until the results are displayed on the screen, as compared to at least thirty minutes for the above listed methods. Additionally, compared to other techniques developed for the same purpose such as MRI^6^, PET^32^ or exogenous fluorescence guided endoscopy^33^, our tool does not require the use of any external agents, thus simplifying the work and limiting any biased classification. As for the techniques based on intrinsic signals such as OCT^34^, or intraoperative ultrasound^35,36^, these simply act as imaging tools that demand the interference of an expert to fully interpret the results. By focusing the work on TPEF, this method gave us the possibility to combine imaging modalities to quantitative measurements to reach unsupervised discrimination in real-time.

In conclusion, this preliminary study highlights the interest of a multimodal two-photon excitation tool to guide intraoperative delineation of tumors’ margins. It may be useful to tailor intraoperatively the surgical resection of malignant brain tumors in addition to brain mapping. In time to come, the present study will be extended to a larger cohort that includes different brain tumor subtypes. This study will address the question of whether the quantitative optical markers studied here can be applied to infiltrating brain tissue located at tumor margins. The final challenge will be to translate the endomicroscope into the operating room and to be approved for *in vivo* clinical studies.

## Materials and Methods

### Samples

This prospective longitudinal study was conducted at a tertiary referral neurosurgical center for brain tumor patients, between March 2015 and May 2017. The human research institutional review board of the Sainte-Anne Hospital – University Paris Descartes (CPP Ile de France 3, S.C.3227) approved the study protocol. All methods were carried out in accordance with relevant guidelines and regulations. An informed written consent was obtained from all patients prior to enrollment. Twenty-five individuals (25 patients, 13 males, 12 females; mean, 51.2 ± 15.2 year-old; range, 19–69 year-old) were included. Fresh human tumor brain tissue specimens (n = 18 from 18 individuals) were obtained from the planned surgical margin surrounding the tumor core (10 metastasis samples, originating from thyroid, larynx, oesophagus, colon and otorinolaringologia carcinoma, bronchial and mammary adenocarcinoma and heel melanoma, summed up in Table 1; and 8 glioblastoma samples, GBM). The gross location of each specimen was recorded intraoperatively with MRI-based neuronavigation (BrainLAB, AG, Feldkirchen, Germany). Control brain tissue specimens (7 patients with no history of brain cancer) were obtained during surgical removal of drug-resistant mesial temporal lobe epilepsy.

**Table 1:** Origin of the metastasis tissues used in this study.

Metastasis (n = 10)	Origin	Number
	*Thyroid carcinoma*	1
	*Larynx and esophagus carcinoma*	1
	*Bronchial adenocarcinoma*	2
	*Mammary adenocarcinoma*	1
	*colon carcinoma*	2
	*Heel melanoma*	1
	*Otorinolaringologia carcinoma*	1

Fresh samples in excess to what was needed for routine histopathological diagnosis were obtained directly from the operating room. Half of each fresh sample under study was sent to histopathology (solution of serum Physio, ambient temperature, black box), where it was formalin-fixed (4% paraformaldehyde), paraffin-embedded, stained with H&E for histopathological analysis, and digitized using Digital Slide Scanner NanoZoomer 2.0 (Hamamatsu Photonics K.K, Hamamatsu, Japan). The other half was carried to the PIMPA platform under similar conditions (solution of serum Physio, ambient temperature, black box) and imaged with TPEF, SHG, FLIM and spectral imaging without tissue fixation. No specimen was excluded from TPM or histopathological analysis. Samples were 1.25 ± 0.45 cm (range, 0.5–2.0) in size and 3.36 ± 1.05 mm (range, 2–5) thick. TPEF, SHG, FLIM and spectral imaging were recorded sequentially without tissue processing within a mean time interval of 1 ± 0.2 s (range, 0.8–1.2 s).

### Correlation between TPM and histopathology

We initially collected 25 paired TPM and H&E images from 25 samples (10 metastasis, 8 glioblastomas, 7 controls). A senior neuropathologist performed an initial histopathological analysis, while blind to the TPM results. No specimen was excluded due to lack of histopathological representativity. To control for intra-observer bias, we presented a Web-based survey of TPM images (png file of the superimposed image of TPM and SHG response) to two senior neuropathologists six months after the initial H&E based diagnosis pertaining to 25 randomly selected patients (control, n = 7, GBM, n = 8, metastasis, n = 10).

They were asked to classify the sampled tissue in four categories: (1) GBM; (2) metastasis; (3) healthy tissue; or (4) unclassified. The neuropathologist had access to clinical data typically available along with the TPM images. This includes patients’ age, gender, clinical presentation, tumor location, and the pre-operative MR images.

The neuropathological blind analysis of TPM images and its corresponding ability to discriminate between control, GBM, and metastasis tissues was evaluated using the classification properties defined as the sensitity, specificty and the accuracy of a diagnostic test, respectively following Eqs 1–3.

1 $${S}_{e}=\frac{TP}{TP+FN}$$

2 $${S}_{p}=\frac{TN}{TN+FP}$$

3 $$Acc=\frac{TN+TP}{TN+FN+TP+FP}$$

TP = True Positive, FP = False Positive, TN = True Negative and FN = False Negative. Their signification depended on:

- Discriminating tumor tissues (GBM, metastasis) from control tissues: TP = tumoral tissue classified as tumoral, FP = control tissue classified as tumoral, TN = control tissue classified as healthy and FN = tumoral tissue classified as healthy.
- Discriminating GBM tissues from metastasis tissues: TP = metastasis classified as metastasis, FP = GBM tissue classified as metastasis, TN = GBM tissue classified as GBM and FN = metastasis classified as GBM.

### Fluorescence and SHG image acquisition

This study was conducted on the multimodal two-photon microscope at the PIMPA (multiphotonic imaging platform for small animals) platform at the IMNC laboratory, Orsay, France. A Mai Tai DeepSee Ti:Sapphire laser source with automated dispersion compensation was used for two-photon excitation. The source’s average power was 2.4 W at 800 nm excitation and was tunable from 690 nm to 1040 nm. The repetition rate of the laser source was 80 MHz and the output pulse duration was set to 70 fs. The laser was combined to a confocal and multiphoton microscope, the TCS SP8 MP (Leica Microsystems, Germany) and was controlled through the Leica software, Symphotime x64. Different visible excitation diodes were also included in the setup, including the 405 nm excitation wavelength. Two remarkably sensitive non-descanned hybrid detectors (Leica, Germany) were used to collect the two-photon fluorescence signal. The collected signal passed through a transparent dichroic filter (680 nm) to laser reflection, then through a second dichroic filter (FF495-Di03-25x36) to direct the light towards the two hybrid detectors. An additional filter (Semrock, FF01-448/20-25, FF01-520/35-25) was placed in front of the detector to define specific spectral bands. Two different water-immersion Leica objectives were used (HCX IRAPO L 20X NA 0.95 and HC PL APO 40X NA 1.1CORR CS2). Images were 512 by 512 pixels in size. The speed scan was 400 Hz and the pixel size was 866.65 nm by 866.65 nm (no zoom factor). The pixel dwell time was 1.20 μm and the frame rate was 0.52 frames per second. Large regions of interest were selected with Leica’s acquisition software.

### Spectral imaging and analysis

The spectrally-resolved fluorescence intensities were detected by a hybrid detector (HyD, Leica, Germany) placed in the confocal head of the microscope piloting the grating and mirror in front of the detector. The spectral resolution was 10 nm, covering the range from 380 nm to 780 nm. A spectral mosaic was acquired on a 3 × 3 image area i.e. a spectral measurement was made for each image of the mosaic then the software merged the information to give its mean fluorescence spectrum.

The spectral excitation-emission matrix was acquired by varying the excitation wavelength and detecting the fluorescence across the whole emission band. The power at the output of the microscope objective was measured using a power meter (Nova II, Ophir, USA) so that the fluorescence spectra were adjusted according to the corresponding excitation power. The acquired spectra were treated with Matlab scripts developed at the IMNC laboratory^30^, where the fluorescence and SHG signal of five endogenous molecules were spectrally decomposed (NADH, FAD, lipopigments, porphyrins I and II).

Five ROIs, each corresponding to 200 μm in diameter were chosen for spectral analysis.

The SHG, NADH and FAD peak intensities were extracted at 890 nm excitation wavelength to calculate two quantitative markers, the SHG peak intensity and the redox ratio (ROx)^25^. The ROx is defined in Eq. 4 as:

4 $$ROx=\frac{NADH}{FAD}$$

### FLIM acquisition

The microscope integrated a FLIM module from PicoQuant (GmbH, Berlin, Germany), in order to acquire fluorescence lifetime imaging. Each ROI (512 × 512 pixels) was also imaged in FLIM mode at a repetition rate of 100 Hz, where the final image was the result of averaging twenty single frames. For each pixel, the fluorescence decay profiles were fit to a mono- or bi-exponential function using the Symphotime software (Symphotime x64 bit, PicoQuant, GmbH, Berlin, Germany) to recover the lifetime values. Ten to fifteen ROIs were selected from different structures observed in the FLIM image. The goodness of fit was assessed by calculating χ2-value as defined in Eq. 5:

5 $${\chi }^{2}=\sum _{i}\frac{{({x}_{i}-{\mu }_{i})}^{2}}{{{\sigma }_{i}}^{2}}\,{\mu }_{i}\,:\,mean,\,{{\sigma }_{i}}^{2}\,:\,variance$$

The criterion for an acceptable fit was having χ^2^-values of around 1.0 (χ^2^ range 0.8 to 1.6). Additionally, the residuals had to be randomly distributed around zero within the intervals 4 and −4. The average lifetime (τ_avg_) for each ROI was measured using Eq. 6.

6 $${\tau }_{avg}=\frac{\sum _{i}{a}_{i}{\tau }_{i}}{\sum _{i}{a}_{i}}$$

### Scoring system

Three quantitative indicators were used: (1) Redox ratio: NADH/FAD, (2) fitted SHG intensity and, (3) the average lifetime, all under an 890 nm excitation wavelength. The three measurements were performed on the same region of interest that could be projected in a 3D space with these values as coordinates. The scatter cloud of a group was fitted by a Gaussian ellipsoid using the mean and the standard deviation as parameters for the covariance so that the ellipse can cover 95% of the total probability mass. The percentage of overlap between the ellipses for each group was then calculated to assess of the performance of such algorithm.

### Statistical analysis

Statistical analyses of the SHG and fluorescence intensities, along with the spectral widths were generated using Matlab (R 2013a), using an ANOVA test for the three tissue groups. This was followed by applying the Bonferroni method to determine which mean values are significantly different within a 95% confidence interval.

The statistical values found in Fig. 6(b–d) were computed using the software R (x64 3.2.0), where the computed p-values < 0.05 were considered to be statistically significant.

## Data Availability

The datasets generated and/or analyzed during the current study are available from the corresponding author on reasonable request.

## References

[1] Hervey-Jumper SL, Berger MS (2016). Maximizing safe resection of low- and high-grade glioma. J. Neurooncol..

[2] Ferguson Sherise D., Wagner Kathryn M., Prabhu Sujit S., McAleer Mary F., McCutcheon Ian E., Sawaya Raymond (2017). Neurosurgical management of brain metastases. Clinical & Experimental Metastasis.

[3] Pallud Johan, Zanello Marc, Kuchcinski Grégory, Roux Alexandre, Muto Jun, Mellerio Charles, Dezamis Edouard, Oppenheim Catherine (2018). Individual Variability of the Human Cerebral Cortex Identified Using Intraoperative Mapping. World Neurosurgery.

[4] Pallud J (2017). Direct electrical bipolar electrostimulation for functional cortical and subcortical cerebral mapping in awake craniotomy. Practical considerations. Neurochirurgie..

[5] Berger Mitchel S., Hadjipanayis Costas G. (2007). SURGERY OF INTRINSIC CEREBRAL TUMORS. Neurosurgery.

[6] Kubben PL (2011). Intraoperative MRI-guided resection of glioblastoma multiforme: a systematic review. Lancet Oncol..

[7] Uluç K, Kujoth GC, Başkaya MK (2009). Operating microscopes: past, present, and future. Neurosurg. Focus.

[8] Croce, A. C. & Bottiroli, G. Autofluorescence spectroscopy and imaging: a tool for biomedical research and diagnosis. *Eur. J. Histochem*. **58** (2014). 10.4081/ejh.2014.2461 PMC4289852 25578980

[9] Papayan G, Petrishchev N, Galagudza M (2014). Autofluorescence spectroscopy for NADH and flavoproteins redox state monitoring in the isolated rat heart subjected to ischemia-reperfusion. Photodiagnosis Photodyn. Ther..

[10] Wu X (2013). Label-Free Detection of Breast Masses Using Multiphoton Microscopy. PLoS ONE.

[11] Yan J (2012). Preclinical study of using multiphoton microscopy to diagnose liver cancer and differentiate benign and malignant liver lesions. J. Biomed. Opt..

[12] Xu, J. *et al*. Identifying the neck margin status of ductal adenocarcinoma in the pancreatic head by multiphoton microscopy. *Sci. Rep*. **7** (2017). 10.1038/s41598-017-04771-w PMC5496940 28676646

[13] Gu M, Bao H, Kang H (2014). Fibre-optical microendoscopy. J. Microsc..

[14] Theer P, Hasan MT, Denk W (2003). Two-photon imaging to a depth of 1000 microm in living brains by use of a Ti:Al_2_O_3_ regenerative amplifier. Opt. Lett..

[15] Takahashi N (2015). Two-photon fluorescence lifetime imaging of primed SNARE complexes in presynaptic terminals and β cells. Nat. Commun..

[16] Xu, C. & Webb, W. W. Reprinted with permission from Journal of the Optical Society of America B, Vol. 13 (3), pp. 481–491 (March 1996). \copyright 1996 Optical Society of America. *Sel. Pap. Multiphoton Excit. Microsc*. **175**, 140 (2003).

[17] Ramanujan VK, Zhang J-H, Biener E, Herman B (2005). Multiphoton fluorescence lifetime contrast in deep tissue imaging: prospects in redox imaging and disease diagnosis. J. Biomed. Opt..

[18] Bastiaens PI, Squire A (1999). Fluorescence lifetime imaging microscopy: spatial resolution of biochemical processes in the cell. Trends Cell Biol..

[19] Chen Y, Periasamy A (2004). Characterization of two-photon excitation fluorescence lifetime imaging microscopy for protein localization. Microsc. Res. Tech..

[20] van Munster EB, Gadella TWJ (2005). Fluorescence lifetime imaging microscopy (FLIM). Adv. Biochem. Eng. Biotechnol..

[21] Perry SW, Burke RM, Brown EB (2012). Two-Photon and Second Harmonic Microscopy in Clinical and Translational Cancer Research. Ann. Biomed. Eng..

[22] Yan Long, Rueden Curtis T., White John G., Eliceiri Kevin W. (2006). Applications of combined spectral lifetime microscopy for biology. BioTechniques.

[23] Skala MC (2007). *In vivo* multiphoton microscopy of NADH and FAD redox states, fluorescence lifetimes, and cellular morphology in precancerous epithelia. Proc. Natl. Acad. Sci..

[24] Chorvat D, Chorvatova A (2009). Multi-wavelength fluorescence lifetime spectroscopy: a new approach to the study of endogenous fluorescence in living cells and tissues. Laser Phys. Lett..

[25] Cicchi R (2010). Time- and Spectral-resolved two-photon imaging of healthy bladder mucosa and carcinoma *in situ*. Opt. Express.

[26] Palmer Scott, Litvinova Karina, Dunaev Andrey, Yubo Ji, McGloin David, Nabi Ghulam (2016). Optical redox ratio and endogenous porphyrins in the detection of urinary bladder cancer: A patient biopsy analysis. Journal of Biophotonics.

[27] Keikhosravi, A., Bredfeldt, J. S., Sagar, A. K. & Eliceiri, K. W. Second-harmonic generation imaging of cancer. in *Methods in Cell Biology* **123**, 531–546 (Elsevier, 2014). 10.1016/B978-0-12-420138-5.00028-8 24974046

[28] Warburg O, Wind F, Negelein E (1927). The metabolism of tumors in the body. J. Gen. Physiol..

[29] Provenzano PP, Eliceiri KW, Keely PJ (2009). Multiphoton microscopy and fluorescence lifetime imaging microscopy (FLIM) to monitor metastasis and the tumor microenvironment. Clin. Exp. Metastasis.

[30] Haidar DA, Leh B, Zanello M, Siebert R (2015). Spectral and lifetime domain measurements of rat brain tumors. Biomed. Opt. Express.

[31] Ibrahim A (2016). Characterization of fiber ultrashort pulse delivery for nonlinear endomicroscopy. Opt. Express.

[32] Ibrahim A (2016). Spectral and fluorescence lifetime endoscopic system using a double-clad photonic crystal fiber. Opt. Lett..

[33] Fink JR, Muzi M, Peck M, Krohn KA (2015). Continuing Education: Multi-modality Brain Tumor Imaging – MRI, PET, and PET/MRI. J. Nucl. Med. Off. Publ. Soc. Nucl. Med..

[34] Huang Z (2017). Fluorescence-guided resection of brain tumor: review of the significance of intraoperative quantification of protoporphyrin IX fluorescence. Neurophotonics.

[35] Böhringer HJ (2006). Time-domain and spectral-domain optical coherence tomography in the analysis of brain tumor tissue. Lasers Surg. Med..

[36] Unsgaard G., Rygh O. M., Selbekk T., Müller T. B., Kolstad F., Lindseth F., Hernes T. A. Nagelhus (2005). Intra-operative 3D ultrasound in neurosurgery. Acta Neurochirurgica.

